# “Let’s Not Have the Perfect Be the Enemy of the Good”: Social Impact Bonds, Randomized Controlled Trials, and the Valuation of Social Programs

**DOI:** 10.1177/01622439211042083

**Published:** 2021-09-07

**Authors:** James W. Williams

**Affiliations:** 1 Department of Social Science, York University, Toronto, Ontario, Canada

**Keywords:** social impact bonds, randomized controlled trials, methodologies, methods, markets/economies

## Abstract

This article uses the case of “social impact bonds” (SIBs) to explore the role of social science methods in new markets in “social investment.” Pioneered in the UK in 2010, SIBs use private capital to fund social programs with governments paying returns for successful outcomes. Central to the SIB model is the question of evaluation and the method to be used in determining program outcomes and investor returns. In the United States, the randomized controlled trial (RCT) has been the dominant method. However, this has not been without controversy. Some SIB practitioners and investors have argued that, while this may be the perfect tool, the need to grow the SIB market demands a more pragmatic approach. Drawing from a three-year study of SIBs, and informed by Science and Technology Studies (STS)-inspired work on valuation and the social life of methods, the article explores RCTs as both a valuation technology central to SIB design and the object of a micropolitics of valuation which has impeded market growth. It is the relationship between, and the politics of, evaluation and valuation that is a key lesson of the SIB experiment and an important insight for future research on “social investment” and other settings where methods are constitutive of financial value.

## Introduction

A “beautiful little jewel box.” That was how a panelist at the 2016 Social Capital Markets (SOCAP) conference described randomized controlled trials (RCTs). The comment was made by Lara Metcalf, an investment banker turned social finance specialist, in reference to the role of RCTs in a new model for funding social programs: the social impact bond (SIB).^1^ First piloted in the UK in 2010, a SIB uses private capital to fund a social program or service with governments providing a return depending on the degree of success. From the outset, a key question for these projects was how “success” should be defined and evaluated. While early SIBs in the UK used a variety of methods, in the United States, the focus has been on RCTs (Milner and Walsh 2016). However, as the US market has evolved, RCTs have come to be viewed as more of a liability, an ideal to be pursued in a perfect world but one that is impractical slowing project development and impeding market growth. It was this more critical view which informed the SIB panel at the SOCAP conference with panelists drawing a stark contrast between the epistemic idealism of RCTs and the market pragmatism required to grow the SIB market. The same individual who described RCTs as a “beautiful little jewel box” complained that they were a barrier to “doing things at scale.” A second panelist compared RCTs to a “Maserati,” an unnecessary luxury compared to the more practical “Camry or Subaru,” while a third argued that relying on RCTs was akin to letting the perfect be the enemy of the good, “I don’t think we should let the perfect be the enemy of the good…. While an RCT gives as close to perfect information as we can get…getting there has real costs, both financial and otherwise” (SOCAP 2016).

This article examines these shifting views around the role of RCTs in the SIB experiment and what this debate reveals about the relationship between *evaluation* and *valuation* and the place of social science methods in constructions of “social value” (Barman 2016). Informed by the results of a larger, three-year study of SIBs in Canada, the United States, and the UK, and drawing from the social studies of valuation (Muniesa 2014) and the social life of methods (Law, Ruppert, and Savage 2011), the article explores how RCTs are not only a method for determining the results of SIB-funded programs but also a device for valuing these projects with the epistemic features of the experimental design translating into a distinct form of financial value. It is by virtue of this valuation work, and the particular type of “value” they perform, that RCTs have become an object of debate championed by some but opposed by others who have a different form of “value” and thus method in mind. In teasing out the dual role of RCTs as a *valuation technology* and the embodiment of a politics or *micropolitics of valuation*, the article both highlights the struggles and limits of the SIB experiment and sheds light on the relationship between evaluation and valuation as a key aspect of larger markets in “social investment.” The analysis of the valuation work performed by RCTs also offers a useful bridge between the literatures on evaluation and valuation, revealing how methods and values are mutually constitutive with important implications for research both within and beyond the social sector. Having outlined the basic argument, the article begins with a more fulsome account of the SIB experiment and how questions of evaluation and valuation have shaped this enterprise.

## SIBs and the Challenge of (E)Valuation

In March 2010, the UK Ministry of Justice announced what was to be the pilot of a new model for funding social programs. Termed a “social impact bond,” the idea was that private investors would provide upfront capital for a consortium of social service providers to work with offenders released from Peterborough prison. If the program succeeded in lowering rates of reoffending, investors would receive a return paid by the government based on the cost savings from reduced demand on the criminal justice system. If the program fell short of its targets, investors would lose their principal and forego any returns. This model was seen to provide benefits to all parties involved. Governments could secure funding for social programs while avoiding the upfront costs and risks of these investments. Social service providers, many of them charities, could access longer-term and more flexible funding. And investors could realize both a financial and a social return, exactly the kind of “blended value” proposition heralded by advocates of the “new social economy” (Bolton and Savell 2010). Buoyed by these perceived benefits, the SIB model quickly gained traction with projects soon being developed in Canada, Australia, the United States, and Europe across a range of policy areas including criminal justice, child welfare, homelessness, employment, and education. By July 2020, the SIB market had surpassed 200 projects worldwide (Gustafsson-Wright and Osborne 2021).

### Evaluating the SIB Experiment

As the slate of projects has grown, so too has the number of commentaries and critiques. Much of the focus of this work, particularly in the academic literature, has been on the perceived flaws of the SIB model and the failure of early projects to deliver on key promises around program innovation, government cost savings, and risk transfer (see Fraser et al. 2018). Concerns have also been raised around the implications of SIBs for the social sector with the model viewed as a further iteration and indeed intensification of neoliberal and market logics and a sign of the growing “financialization” of social services and public policy (Warner 2013; Dowling 2016; Sinclair, McHugh, and Roy 2019). There is certainly merit to these critiques as SIBs, without question, embody neoliberal and financial logics. However, what has been overlooked is how the SIB experiment has actually fared in practice and the work involved in developing projects and building the SIB market. Transforming social sector nonprofits into viable investments with clear returns and risk profiles is no easy feat and hinges on the ability not only to assign an economic and financial value to what is ostensibly a social good but also to develop a value proposition that is convincing to all parties involved (i.e., government, providers, and investors). At the heart of SIBs is thus a question of value and a distinct challenge of valuation.

In the handful of accounts that have acknowledged this question of value and sought to provide a more finely grained account of SIB design, the focus has been on financial and accounting tools, what Cooper, Graham, and Himick (2016, 73) refer to as “economic technologies,” and their role in transforming “outcomes” into cash flows (Chiapello 2015; Berndt and Wirth 2018; Neyland 2018). Little attention has been devoted to how program outcomes are determined in the first place, a question of *evaluation* that is fundamental to the SIB model, given that it is the measurement of outcomes that dictates whether or not, and how much, investors will be paid. As explained in one report, “With investment returns riding on the demonstration of program results, determining the best approach to measuring success is a critical challenge for many PFS stakeholders” (Milner and Walsh 2016, 1). Evaluation in a SIB context is thus “high stakes” (Savell and Heady 2016, 4).

These stakes are especially evident in the United States where evaluation has emerged as a key point of debate. As revealed by the comments of our SIB panelists, battles have been fought between those who favor RCTs as the best measure of program success and those who advocate for less rigorous, more practical alternatives. This very tension is noted in a report by a SIB intermediary which observed that, “Debate, particularly in markets like the US, is increasingly polarized among those that maintain that only randomized control trials (RCTs) will do, and those that advocate less intensive approaches in order to accelerate the market” (Savell and Heady 2016, 2). Ultimately, the RCT debate begs the question of exactly how evaluation and the choice of method inform the valuation of these projects, and whether it is *because of* the valuation work they perform and the type of “value” they produce (vs. simply their “intensity”) that RCTs have become a magnet for critics. The debate also points to the contested nature of SIB development and the internal tensions within the “SIB space,” conflicts that have been overlooked in the SIB literature to date. What requires further exploration is thus the distinct nature and politics of RCT-based valuations and their impact on the SIB enterprise.

### Bridging the Evaluation/Valuation Divide

The analysis of RCTs and the valuation of SIBs are aided by, and seek to bridge, two social science literatures. The first is the social studies of economization and valuation, which is concerned with the creation of new markets and how economic value is ascribed to noneconomic goods. Inspired by insights from science and technology studies applied to the world of economics, finance, and markets, a core tenet of this work is that economic value is not intrinsic to objects or practices but rather is actively produced and performed (Callon and Muniesa 2005; Muniesa 2014). Central to this process of value creation is the enactment of sociotechnical networks and the mobilization of tools and devices which disentangle objects from their natural contexts and reconstitute them within new calculative spaces, a process of abstraction, quantification, and standardization that is critical to making things valuable (Callon and Muniesa 2005; Caliskan and Callon 2010; Muniesa 2014). Business models are one such valuation device representing not only a calculative tool but also a type of narrative that is future-oriented and promissory helping to engage and align different groups of actors (Doganova and Eyquem-Renault 2009; Doganova and Muniesa 2015).

While they have received limited attention in the valuation literature to date, research methods are another key valuation device. One of the few accounts to conceive of methods, and RCTs specifically, through this lens is work by Helgesson and colleagues which explores how the choice of research design in the context of pharmaceutical trials is informed by, and contributes to, different articulations of value (Helgesson, Lee, and Lindén 2016; Helgesson and Lee 2017). Focusing specifically on the transition from traditional RCTs to more flexible alternatives (such as adaptive designs), the authors argue that these new designs are rooted in a different conception of value, one that privileges adaptability and speed over epistemic rigor and objectivity (see also Montgomery 2017). The notion that research methods both enact and reflect different articulations of “value” in this way also resonates with work on the “social life of methods” which explores how methods are both constitutive of, and constituted by, the social world, what is referred to as the “double social life” of methods (Law, Ruppert, and Savage 2011; Ruppert, Law, and Savage 2013). These authors describe a similar transition from traditional methods such as surveys toward new digitized, data-based techniques, further evidence of a growing methodological pragmatism, while focusing on the social causes and consequences of this shift. Together, these accounts highlight the stakes underlying the choice of research design and how different designs embody and produce different logics and forms of value.

Another relevant body of work is the social science literature on RCTs. While much of this work has focused on the use of RCTs in a biomedical context including evidence-based medicine (Marks 1997; Timmermans and Berg 2003) and pharmaceuticals (Will 2007; McGoey 2010; Kelly and McGoey 2018), scholars have also examined the expansion of RCTs into the world of social and public policy particularly in the context of international development (Abdelghafour 2017; Donovan 2018; Rayzberg 2019; de Souza Leão and Eyal 2019). Several of these accounts explicitly invoke an STS perspective noting how the spread of RCTs has been enabled by particular sociotechnical networks and framing RCTs themselves in terms of a sociotechnical assemblage (Abdelghafour 2017; Rayzberg 2019). And yet, while capturing their social dimensions and technical complexity, these accounts have little to say about the valuation work performed by RCTs—that is, how they produce particular forms of value.

### Framing the SIB Enterprise

Building on these respective literatures, SIBs may be viewed in terms of a distinct process and practice of valuation. SIB-funded projects are rooted in the creation of a sociotechnical network composed of a whole host of calculative devices, models, and methods through which the value of these projects is constituted or performed. The development of this sociotechnical network also requires the enrolment of the essential partners to these deals (e.g., government, investors, and providers), each of whom has different worldviews, interests, and forms of “value” in mind. In the context of the RCT debate, the question is exactly how the selection of the RCT as the appropriate evaluation methodology informs the valuation work and the type of “value” produced through these projects—that is, how the features of RCTs as a complex “socio-technical device” (Abdelghafour 2017), or what I later describe as a *valuation technology*, shape the SIB value proposition and inform the politics of this space.

The remainder of the article explores the work and politics of (e)valuation underlying the SIB experiment, drawing from the results of a larger, three-year study of SIBs in Canada, the United States, and the UK. This study sought to move beyond published reports and case studies of individual projects and to focus instead on the experiences of those directly involved in, and impacted by, these projects. In all three countries, the work of SIB development has fallen to a handful of specialist advisory firms established with the express purpose of launching projects and building the SIB market.^2^ The study started with these SIB practitioners and then moved outward, focusing on other key actors in the SIB ecosystem including government officials, social service providers, investors, and philanthropists. In total, 196 semistructured interviews were conducted between May 2016 and July 2019, primarily in the cities of Toronto, Boston, and London. These interviews yielded a very different view of the SIB enterprise. Contrary to the public image of a market on the rise, SIB practitioners expressed frustration with the slow growth in the number and scale of projects and cited a host of challenges that had impeded their efforts to build the SIB market. It was in describing these challenges that RCTs emerged as a common point of contention, particularly for US respondents, and it is the conversations around the merits and drawbacks of RCTs that inform the discussion to follow.

## Valuing Social Programs: RCTs as a Valuation Technology

At the heart of the SIB model is the notion that the work of social service nonprofits—the charitable organizations providing housing to the homeless, reoffending programs for those released from prison, and family support for children at risk of going into state care—possesses an economic value which may be captured, monetized, and converted into savings for governments and returns for investors. As explained by a former investment banker and senior member of the US SIB space,

The beauty of [SIBs] lies in the notion that social impact…not only…[has] economic value…you can monetize that economic value and create a cash flow from it. I know from my days in investment banking, you give me a cash flow that I can identify, lock up, and secure, I can finance anything. (Pinakiewicz 2014)

The idea that nonprofits generate economic and financial value in this way involves a shift in how social sector providers are themselves assessed. Traditionally, providers have been compensated on the basis of their “outputs” as indicators of agency activity such as program enrolments and completions (e.g., the number of individuals who complete a job training program). In contrast, SIBs are built around “outcomes” as measures of the positive change produced by these efforts (e.g., the number of individuals employed as the result of a job training program). Whereas “outputs” are transactional and retrospective in nature, “outcomes” are transformational and future-oriented. Thinking in terms of “outcomes” is critical to the SIB model and its financial logic as this frames nonprofit work in terms of its *future* impact and value thus allowing for social programs to be turned into a type of *asset*—that is, an investment in the present that yields value in the future (Birch and Muniesa 2020).

And yet, the value of SIB-funded programs depends not only on the “outcomes” that are produced but also on the extent to which they are additive—that is, they represent *net gains* relative to the status quo or what would have been produced in the absence of the program. While securing employment for the graduates of a job-training program is a positive outcome, the real value of this type of program depends on whether these same individuals would have been employed without it. The value of “outcomes” is thus relative and hinges on the construction of a counterfactual or what Ehrenstein and Muniesa (2013) refer to as a form of “counterfactual display” where “two future states of the world—one with the project and one without it—are played against each other” and the value of the project is “derived from that interplay” (p. 162). It is the difference, or what SIB practitioners often refer to as the “delta,” between the intervention and the status quo that is constitutive of the value of these programs.

The question, then, is exactly what this “counterfactual display” should look like—that is, what rules should inform or govern the construction of this “prospective reality” (Ehrenstein and Muniesa 2013, 180). Early reports on SIBs were clear that investors should only be compensated for net benefits but were less prescriptive regarding the specific method to be employed (Bolton and Savell 2010; Mulgan et al. 2011). The first SIB in Peterborough used a quasi-experimental design in which the recidivism rate for program participants was compared to a national sample of offenders matched using propensity score matching. In the very next project, a child welfare SIB in Essex, a live counterfactual was deemed unnecessary with payments based on gains relative to an historical baseline (Savell and Heady 2016).

As the SIB model was exported to the United States, it was RCTs specifically that emerged as the preferred method, with seven of the first eleven projects drawing from RCT-based evaluations (Milner and Walsh 2016).^3^ In each case, the rules of “counterfactual display” followed the conventions and dictates of the experimental design. For example, in two early criminal justice projects, a juvenile justice program in Massachusetts and a program for adult offenders in New York State, prospective participants were randomly assigned to a program and a control group with payment based on statistically significant improvements (defined as a reduction in the number of “bed days” of incarceration) in the program group relative to the control. As with most applications of the experimental method in a social policy context, these evaluations lacked other essential features of the classic RCT design including double-blind assignment and a controlled setting. They are thus more accurately viewed as “field experiments” (Rayzberg 2019) but are nevertheless described by SIB practitioners as “RCTs.”

While a variety of factors contributed to the embrace of RCTs in the United States, including the stronger tradition of RCT-based evaluations and the influence of key actors such as the Harvard Government Performance Lab (GPL),^4^ the main driver has been the perceived epistemic attributes of RCTs and how they are seen to translate into a specific form of economic value. RCTs allow for the translation of the complex work of social programs into a precise measure of effect size, a form of quantification necessary for the monetization of programs and the calculation of savings and returns. And yet, RCTs are not unique in this ability. Quasi-experiments are also able to provide these kinds of quantitative renderings. What is distinct about RCTs is their degree of rigor and perceived power of attribution. Based on the epistemic features of the experimental design, most notably the element of randomization, they are seen to produce the most rigorous assessments of *both* additionality (the outcomes produced by programs relative to the status quo) *and* causality (the extent to which outcomes are attributable to the program in question). In contrast, less rigorous designs are more susceptible to extraneous influences and can only provide indications of “outcomes” which are less valuable, given that they lack the certainty and attributability of “impacts.” The power of RCTs as a valuation device derives from its *degrees* of counterfactual rigor, which correspond to *real differences* in economic value. As one SIB practitioner explained, “outcomes” are worth less than “impacts” because “you don’t have the counterfactual to the same degree. And it’s not that outcomes are bad. They are of a *different nature* and they are *worth different things*” (US respondent #23). Informed by the most rigorous rules of counterfactual display, the results of RCTs are ultimately worth more because they possess distinct qualities (Callon, Méadel, and Rabeharisoa 2002), which equate to greater economic value.

By virtue of their epistemic rigor, RCTs also possess a symbolic value (Donovan 2018; Kelly and McGoey 2018). Payment on the basis of RCTs allows governments to claim that they are paying investors only for real “impact” validated using the most rigorous methods possible. In the words of a staffer at a foundation that has been a key booster of SIBs, “One of the reasons that we think RCTs are so important…was this need to ensure that outcomes were rigorously evaluated and the government was paying private investors…based on the impact of programs and not just on what would have happened anyways” (US respondent #8). RCTs thus offer a legitimacy cover for governments sensitive to the political risks and potentially tricky optics of paying investors to deliver social programs.

In a SIB context, RCTs function in a manner that closely resembles the devices examined by valuation scholars. Like business models, RCTs create a “calculative space” (Callon and Muniesa 2005, 171) through which the complexities of social programs are simplified and distilled into discrete, linear, and quantitative effects. RCTs are also performative, enacting and constituting the juxtaposed realities of the program and its counterfactual. And they are rooted in a particular kind of imaginary with the control group representing a proxy for the world without the program and thus an alternative future for program participants, “The assumption with an RCT is you find someone who came through the same referral pathway as Jane and you put that person in the other group and that person is Jane without this intervention” (US respondent #5).

And yet, in performing these functions, RCTs are more than simply a tool or “device.” Rather, they are more accurately described as a valuation *technology*, an assemblage of different components and moving parts, including design features and protocols (e.g., randomization), statistical rules and procedures (e.g., significance testing), data flows and computer software, and epistemic norms and interpretive conventions. This echoes Abdelghafour’s (2017) description of RCTs as “a complex device articulating techniques (e.g., data collection, logistics, computing, communication), theoretical corpuses (experimental sciences, social engineering, economics, inferential statistics), and material equipment (GPS, questionnaires, software) to produce a form of knowledge eventually materialized in academic papers, policy briefs, books and speeches” (p. 241). It is these very elements that are constitutive of the rigor and thus the value of RCTs. They are also a key reason why, as the SIB market has evolved, not everyone has remained convinced of the merits of the RCT-based value proposition.

## RCTs and the Micropolitics of Valuation

As SIB practitioners gained more experience in developing projects, they quickly realized that RCTs came with a host of challenges. This is perhaps not surprising. RCTs have been controversial in nearly all settings in which they have been introduced with many examples of how they have adversely impacted existing relationships and ways of working (e.g., Marks 1997). However, what is interesting in a SIB context is the extent to which these challenges stem specifically from the role of the RCT as a valuation technology and the tensions between the epistemic features of RCT-based designs and the financial logics animating SIBs. Although critics recited many of the common complaints about RCTs—they are expensive, complex, and raise potentially troublesome ethical issues—it was less the logistical barriers and more the valuation work performed by RCTs that was the real problem. While valued by government, there are three specific ways in which RCTs undercut the value of SIBs from an investor perspective thus revealing the distinct politics of “value” in this space: (1) “evaluation risk,” (2) project timelines and the cost of capital, and (3) performance management.

### “Evaluation Risk”

The most common concern around RCTs is that they add to the risk of what are already viewed as risky investments. As noted by a UK investor, “paying against outcomes linked to an RCT…takes what is already quite a risky proposition with a lot of factors that you can’t really control and it squares them” (UK respondent #55). From the perspective of a US investor, the problem is not simply that RCTs are risky but that they create a new *form* of risk, “[The RCT] adds a…risk that in the early days we didn’t think of, which is *evaluation risk* which basically is the risk the structure and process of the evaluation will actually impact the results that are observed” (US respondent #55). One way that RCTs can impact results is through the conventions of significance testing and requirements around statistical significance. In order for investors to be paid, programs must yield not only positive but also “statistically significant” results. A key question is exactly where the threshold for statistical significance should be set. Several respondents suggested that governments (or advisors to government) were pushing the 95 percent threshold.^5^ A common standard for academic publications, this reflects an effort to minimize the risk of false positives—that is, the odds of producing a positive effect that did not actually exist. The problem is not simply that this is deemed to be an overly high bar but that it is seen to benefit the government which faces lower odds of paying for a false positive, while exposing investors to a greater “risk of false negatives” (US respondent #55) and thus not being paid for real results. By virtue of their epistemic features, RCTs thus introduce sources of *statistical* risk which are born by different groups and which are seen to result in greater *financial* risk for investors. Acknowledging this dynamic, a SIB practitioner argued that government rather than investors should bear this risk, “government ought to own the risk of a false positive. You are paying now on, you are trying to avoid [false positives]. Maybe you should try to avoid [a false negative]. Right? And government should own that risk” (US respondent #30). And yet, this is difficult to square with the whole premise of SIBs, which is that risk is *owned* by investors.

Another issue associated with RCTs, and considerations of statistical significance, is pressure on referrals. All SIB-funded projects depend on referrals of prospective clients from other agencies and providers, and maintaining a certain level and flow of referrals is critical to producing monetizable “outcomes” and meeting performance targets. By requiring providers to populate both a program and a control group, RCTs effectively double the number of referrals that are needed. Moreover, given that statistical significance is sensitive to sample size, each group must also be large enough (100–200 is often used as a benchmark) to produce statistically valid results (Bolton and Savell 2010; C. Fox and Albertson 2011). The result is a significant strain on referrals, which are already a source of frustration in many SIB projects.^6^

Struggles around referrals have also impacted how investors evaluate these projects. A respondent who had invested in several SIBs indicated that in diligencing projects, they now pay more attention to potential issues around referrals including the proportion of the target population that must be enrolled based on the required sample size,

We’ve started to look at…the estimated size of the eligible population…and look at how many people need to be enrolled in the project in order to reach a certain sample size and have sufficient statistical power for the evaluation…So we’ve come up with some rough benchmark percentages just to say at the beginning of a project how risky is the enrolment piece of it. (US respondent #60)

“Enrolment and execution risk” is thus another aspect of the “evaluation risk” of RCTs, a risk which is especially challenging given that it is “divorced from any actuarial basis” (US respondent #3) and is thus difficult to capture and quantify.

### Project Timelines and the Cost of Capital

The statistical parameters of RCTs are also significant in another respect. Given that most providers can only handle a certain volume of clients at any one time, multiple cohorts may be needed to accumulate the sample size required for statistical significance. The result is longer project timelines, which create two issues around SIB valuations. First, while governments may be content with longer time horizons, especially if this allows for more rigorous evaluations, investors are less enthusiastic as their capital is tied up and at risk for longer periods of time. As noted by a SIB specialist in reference to a criminal justice project, RCTs create a disconnect in terms of the timelines for these projects, “by the time you know the results of recidivism…you are five or six years down the line. That’s a long time to tie up capital…. So there’s this different time horizon that the parties have that is driven by the RCT” (US respondent #30).

Second, these longer timelines also increase the cost of projects, a function of how returns are calculated. As with any investment, SIB returns are assessed based on their “net present value.” This is an accounting convention that takes into account the “time value” of money—that is, the notion that money is worth more in the present than in the future, given the uncertainty and opportunity costs associated with investing (Doganova 2018). To compensate for this time value of money, a discount rate (usually between 2 percent and 7 percent) is applied to expected returns yielding the investment’s net present value. To be financially viable, the returns from SIBs must exceed these discount rates. Here time, once again, becomes an enemy. The longer a project runs, the greater the discount that must be applied and the higher the returns that will be expected by investors. In extending the time lines of projects, RCTs thus also increase their costs, making it more difficult to design economically viable projects especially as higher returns erode public cost savings, a clear sign of the disconnect between the statistical properties of RCTs and the financialized forms of valuation (Chiapello 2015) underlying SIBs.

### Performance Management

Finally, RCTs pose a distinct challenge to one of the most critical aspects of SIB projects: performance management. Once projects are launched, project managers are responsible for monitoring, in as close to real time as possible, the status of programs identifying areas where performance is falling short and introducing course corrections as required. Particular attention is devoted to indicators such as referrals, enrolments, and completions, which are deemed to be correlated with, and predictive of, future outcomes and returns. The ability to manage programs in this way is critical to ensuring that projects remain on track with performance management described as “the secret sauce of PFS” (US respondent #10), the place “where the magic happens” (UK respondent #55).

And yet, this style of management conflicts with RCT-based evaluations. The absence of regular data on the performance of the control group makes it difficult to manage toward specific outcomes. As explained by a UK SIB specialist, “it’s quite difficult to deliver when you don’t know how well you’re doing. We didn’t know how well we were doing until retrospectively we were measured” (UK respondent #31). In speaking to the importance of performance management, a US respondent likened RCTs to a “black box,” “it’s really about performance management and so getting that real-time feedback is really important. And the RCT doesn’t lend itself to that. It’s like a black box that you open up one day” (US respondent #55). As noted by valuation scholars, value is not something that is prescribed in advance but rather is actively managed and performed (Birch and Muniesa 2020). RCTs interfere with this valuation work, limiting the ability of SIB practitioners to bring the reality of projects back into line with the projections of the model and to “fix the future as it unfolds” (Montgomery 2017, 236). The result is a critical tension not only between the epistemic and financial features of SIBs but also in how value is *performed* in these projects.

### The Micropolitics of Valuation

As these pitfalls of RCTs have become more evident and better understood, the question of evaluation has emerged as a clear point of tension in the SIB space. One US SIB practitioner suggested that evaluation “always comes up” as a point of tension,

You have governments who say, “we don’t want correlation, we really want to understand whether this program is causing these outcomes and the only real way to do that is through a randomized controlled trial”…. And then you have investors who…are thinking about how…this will impact how they will get repaid. (US respondent #25)

Another SIB practitioner echoed this sentiment, “That is absolutely one of the biggest points of tension in PFS…. There’s a lot of opposition to RCTs” (US respondent #20). Respondents also provided examples of where battles had been fought over RCTs. In one project, the service provider and intermediary did not want to do an RCT, but the government and their advisors insisted. According to the provider, “In order to get the whole deal, we had to do an RCT” (US respondent #49). Another provider reported a similar experience describing how they got into a “battle over randomized control” that was almost a deal-breaker (US respondent #22). And yet, there are signs the critics may be winning this battle. Several respondents indicated that the US has started to move away from RCTs, “if you look at how people are doing PFS contracts now, there’s a shift away from doing RCTs for payment purposes which I think is smart” (US respondent #68). A prominent SIB investor agreed, “my sense…is the tide is turning which I think is a good thing” (US respondent #55).

The question that arises from this debate is not whether RCTs are ultimately good or bad. In describing RCT-based projects as “beautiful,” SIB practitioners do more to valorize than critique experimental designs. Conversely, the flaws of RCTs, and their departures from the imagined epistemic ideal, are well-documented (Will 2007; Deaton and Cartwright 2017). Rather, the point is that these diverging views reflect different ways of valuing these investments. They embody different rules of “counterfactual display” and interpretations of how epistemic rigor translates into economic value. For government, “value” depends on net benefit defined relative to a live counterfactual, and the confidence that they are paying investors for real program “impacts” validated using the most rigorous measures possible. For investors and many SIB specialists, “value” is associated with a less rigorous and more retrospective form of counterfactual display involving gains in “outcomes” relative to historical baselines. From this vantage point, the mere fact that a positive outcome has been achieved is of value, given the poor outcomes of the past, “Because *it is a good*…if you’re chronically homeless and we have you in housing for six months, we don’t need to compare that to anything because we know that by definition treatment as usual was not solving that problem” (US respondent #30).^7^ “Value,” for investors, is also tied to the certainty of outcomes and the ability to quantify and mitigate financial risk. RCTs introduce uncertainties that make these projects more difficult to value and impede the ability to manage toward specific outcomes.

Given these tensions around the question of how to “value” SIB-funded projects, RCTs may be viewed as not only a valuation technology allowing for the translation of social programs into investment propositions but also a reflection of the distinct politics underlying this endeavor, what may be described as a *micropolitics* of valuation. Informed by the Foucauldian notion of the microphysics of power (Foucault 1977), and the relational and materialist ontology of Deleuze and Guattari (1987), the concept of “micropolitics” has been deployed across a range of scholarship to describe how power and politics are manifested within, and operate through, the small details of everyday practices, identities, and object relations (e.g., DeHart 2008; N. Fox and Klein 2020). As it applies in a SIB context, the notion of micropolitics helps to capture the distinct form of politics inhering in the *technicalities* of SIB design, in the *technical question* of how to value SIBs and the consequences and tensions that flow from the choice of method. By virtue of their epistemic attributes and features as a valuation technology (e.g., randomization, significance testing), RCTs impose constraints, trade-offs, and unintended consequences that impact the distribution of risks and rewards boosting the value proposition for governments while weakening the case for investors. Returning to Helgesson, Lee, and Lindén (2016), it is true that the choice of research design is informed by, and contributes to, different articulations of “value.” However, what is critical in the case of SIBs is that these decisions are more explicitly politicized with the choice of method representing a key *stake* in competing visions of what SIBs are and how they should be designed and valued.

This notion of micropolitics is helpful in distinguishing the politics of valuation from the more “macro” politics that also inhabit the SIB space, the politics of competing philosophies, normative commitments, and diverging views around the merits of SIBs and the desirability of private investment in social programs. While the latter have certainly impacted the SIB market, it is the former that have posed the more fundamental challenge to the SIB enterprise. The concept of micropolitics is also distinct from the way that politics is often conceived in the valuation literature—that is, in terms of the consequences of market design and the challenges posed from *outside* of the market (Callon 2009). This view is implied, for example, in MacKenzie’s (2009) concept of “subpolitics” where actors outside of the formal political system seek to influence market design. Instead, micropolitics reflects the tensions that exist *within* markets, in the *interior* struggles between different groups of actors, and in the *emergent* politics of models and methods as both objects and stakes of valuation work.

Recognizing these micropolitics of valuation brings us back to the critiques of RCTs with which this article began, critiques that were echoed by respondents in what they described as a clash between an “academic perspective,” a not so thinly veiled reference to the Harvard GPL, and a more “practical” view,

Everybody was trying to be perfect. And I can tell you after six years you can’t be perfect. You have to always be looking at it and saying what is more important to move the needle? Is this about perfection in an academic sense…or is it getting a good project launched?…. You have to look at it in a practical sense sometimes. (US respondent #19)

For another respondent, the influence of the academic perspective, and the privileging of the rigor of RCTs, was a clear case of the perfect becoming the enemy of the good,

People got so enthralled with the early idea of PFS, and a large majority of them coming out of academia, that I think we were a little bit tied to some of these idealistic models or viewpoints of how PFS should work in a perfect world. And I think sometimes perfect became the enemy of the good. And so we can build these beautiful, bespoke little projects that have RCTs…[but this] works against the field because it makes them slow to launch. (US Respondent #45)

RCTs are no doubt challenging to implement, and they have certainly slowed SIB development. However, it is not only the impracticality of RCTs that is the problem but the type of “value” that they invoke and the politics that they embody. The very framing of the RCT debate in terms of the juxtaposition of epistemic idealism and market pragmatism and the aphorism of “the perfect being the enemy of the good” are reflections of, and efforts to intervene in, these politics. They are discursive strategies designed to advance less rigorous forms of (e)valuation, and thus promote a different form of “value,” while scanting the compromises and trade-offs that this satisficing inevitably entails. Ultimately, it is these distinct micropolitics of valuation that help to account for the slow growth of the SIB market not the result of RCT-induced constraints but rather the more fundamental and tractable politics underlying the SIB value proposition.

## Conclusion

Growing interest in how financial capital may be mobilized to address critical social challenges is a legacy of the financial crisis of 2007-08. Central to this effort and the emerging field of “social investment” is the question of how to capture and quantify the “social value” produced by these investments, and the methods appropriate to this task (Barman 2016). SIBs are both an embodiment of and a test case for this vision of social finance and the investability of social programs—and experiment not only in the marriage of social impact and financial returns but also in the role of evaluation and social science methods in these new articulations of social (and financial) value.

Informed by an in-depth account of the SIB enterprise, this article has explored how this question of evaluation has cast a large shadow over the “SIB space.” On the one hand, RCTs have been pivotal to many US projects serving not only as an evaluation tool or a method for gauging the results of projects and determining investor returns but also as a valuation *technology* with the epistemic attributes of the experimental design and the more rigorous rules of counterfactual display performing a distinct form of financial value. On the other hand, RCTs have been the object of an increasingly animated debate. Praised by some for their epistemic virtues, they have been maligned by others for their practical constraints, inherent risks, and overly strict rules of counterfactual display. RCTs thus reflect the distinct politics underlying the SIB enterprise, what has been described as a *micropolitics* of valuation, in which battles around the choice of method reflect different articulations of “value” and ways of valuing these investments. RCTs are not the only reason why the SIB market has struggled (Williams 2020), but the RCT debate exemplifies the unique challenges involved in turning social programs into investable assets and enlisting social science methods in the creation of financial value.

Looking forward and beyond SIBs, this analysis suggests that, in exploring other markets in social investment, we must be mindful not only of the consequences of finance moving into the social sector and the potential for the “financialization” of this space but also of the smaller and more nuanced challenges around the actual work of making this happen, of crafting the valuations upon which social investment depends, and the critical role of evaluation in both enabling and frustrating this enterprise. The case of SIBs also points to the need for further dialogue between the literatures on evaluation and valuation across different contexts with particular emphasis not only on the role of research methods such as RCTs as valuation technologies but also the distinct politics underlying this valuation work.

Finally, while this article has explored how questions of method have informed constructions of value, more work is needed on how shifting forms of value are impacting these methods themselves. Research on pharmaceutical trials has revealed how shifts in the nature of value, specifically the focus on expediency and adaptability over rigor and standardization, have spurred the development of more flexible alternatives to RCTs (Helgesson, Lee, and Lindén 2016; Helgesson and Lee 2017; Montgomery 2017). Similar shifts are occurring in the social sector. As more emphasis is placed on the economic value created by social programs, and the production and maximization of monetizable “outcomes,” evaluation is viewed less as a retrospective assessment of what a program has achieved and more as a way to manage programs in real time based on anticipated *future* outcomes. Thus, rather than RCTs, the focus is increasingly on more flexible, data-driven methods imported from the private sector such as machine learning, simulations, and rapid cycle testing.^8^ These are similar to the transformations described in work on the “social life of methods” (Law, Ruppert, and Savage 2011; Ruppert, Law, and Savage 2013). Building on the notion that methods have a “double life” not only in terms of their relationship with the social but also when it comes to the question of value, the issue for future research is how these new ways of valuing nonprofit work are impacting the choice of method and, in turn, the type of “social value” that these methods (and programs) produce. Given the growing methodological pragmatism within and beyond the social sector, it may not be perfection that we ultimately need to worry about.

## Notes

1. In the United States, these are more commonly referred to as “pay-for-success” projects. However, for the sake of clarity, the term “social impact bond” is used throughout the article.

2. Key social impact bond (SIB) specialists include Social Finance UK, Social Finance US, Third Sector Capital Partners, and the Harvard Government Performance Lab (GPL; formerly known as the Harvard SIB Technical Assistance Lab).

3. Of the four projects that did not include randomized controlled trials (RCTs), two (Rikers Island and Massachusetts chronic homelessness) initially pursued an RCT but eventually opted for nonexperimental designs.

4. The GPL has worked directly with governments in a number of projects often negotiating on their behalf. Respondents suggested that the GPL has been a key booster of RCTs.

5. This threshold is based on “*p* value” which refers to the probability that a result is due to chance. The 95 percent threshold thus equates to a *p* value of .05 and a 5 percent probability that results are due to chance.

6. Both of the early, RCT-based criminal justice SIBs in Massachusetts and New York State struggled with referrals.

7. It is this very logic that underlies the “rate card,” which quickly became the dominant contracting structure for SIBs in the UK. In this case, the government establishes a set menu of “outcomes” that have been priced based on historical performance data. Providers are then compensated for each “outcome” they achieve based on validated administrative data, thus avoiding in-project evaluations altogether.

8. Many SIB advisory firms are themselves proponents of these new methods which they see as the basis for a new advisory market geared toward improving how governments and foundations invest in social programs without having to worry about third-party investors.
